# Single Pontine Relapse Shortly After Hippocampal Avoidance Whole Brain Radiotherapy: A Case Report

**DOI:** 10.1002/cnr2.70323

**Published:** 2025-08-24

**Authors:** Zeinab Dandash, Bassem Youssef, Ali Al Zein, Arafat Tfayli, Toni Tannoury, Lara Hilal

**Affiliations:** ^1^ Department of Radiation Oncology American University of Beirut Medical Center Beirut Lebanon; ^2^ Division of Hematology‐Oncology American University of Beirut Medical Center Beirut Lebanon

**Keywords:** brain metastasis, cognitive decline, hippocampal sparing, SRS, WBRT

## Abstract

**Background:**

Brain metastasis represents the most prevalent form of brain tumors in adults, with a rising incidence resulting from significant advancements in cancer detection and therapeutic interventions. Current treatment protocols advocate for whole brain radiotherapy (WBRT) for patients who are not candidates for either surgical resection or stereotactic irradiation. However, cognitive decline remains a major side effect of this treatment modality. Hippocampal‐sparing WBRT (HA‐WBRT) has been shown to decrease brain toxicity, with the main concern being the probability of developing new brain metastasis in the hippocampal avoidance region.

**Case:**

We report a case of a 73‐year‐old male presenting with multiple brain metastases and treated with HA‐WBRT, who then developed a new single pontine lesion shortly after that was found to be located in an under‐dosed peri‐hippocampal area. We dosimetrically compared the patient's original IMRT plan to three new plans: a standard VMAT plan, an optimized IMRT plan, and an optimized VMAT plan, where optimization incorporated brainstem coverage as a planning objective, resulting in a notable improvement in brainstem dose distribution.

**Conclusion:**

HA‐WBRT poses a risk of peri‐hippocampal metastasis due to underdosing of the upper brainstem that is inherent in HA‐WBRT plans. Planning techniques should focus on optimizing coverage of the brainstem in an attempt to decrease this uncommon occurrence.

## 1 Introduction

Brain metastasis is the most common type of brain tumors in adults, with an incidence four times higher than primary tumors, estimated at 98,000–170,000 cases diagnosed annually in the US [1, 2]. This incidence has been increasing for the past years due to several factors, including improved cancer survival following significant advances in cancer treatment, including targeted therapies, immunotherapies, and chemotherapy. This longer survival allows more time for cancers to metastasize to the brain. A second factor would be the enhancement of imaging techniques, such as MRI, which have improved the detection of smaller and asymptomatic brain metastases that might have been missed previously. A third factor that may have contributed indirectly to the increased brain metastasis incidence is the increased prevalence of breast cancer in recent years, which is among the most common cancers that metastasize to the brain [1, 2, 3, 4].

In 2019, the American Society for Clinical Oncology (ASCO), the Society for Neuro‐Oncology (SNO), and the American Society for Radiation Oncology (ASTRO) developed a joint guideline for the treatment of brain metastasis [5]. This guideline states that surgery is the best option for patients with large brain tumors associated with mass effect, while SRS alone should be offered to patients with 1–4 unresected brain lesions, and in certain conditions 5–10 lesions, who are asymptomatic, do not have small cell carcinoma, and lack effective systemic therapy options. SRS to the surgical cavity should also be offered to patients with 1–2 resected brain metastases [5, 6]. Patients who are ineligible for both surgery and SRS are recommended to have whole brain radiotherapy (WBRT) as a primary treatment.

Despite its role in prolonging survival and managing neurologic symptoms, WBRT can lead to cognitive dysfunction, including learning and memory decline [7, 8, 9]. To attenuate this side effect and improve patients’ quality of life (QOL), a similar but safer approach has been designed, which is the hippocampal avoidance whole brain radiation therapy (HA‐WBRT). This method delivers radiation with high doses to the tumors while sparing the hippocampus, the center of memory and cognition. It is usually offered to patients eligible for WBRT, with no brain metastasis within 5 mm from the hippocampus, and with at least 4 months expected survival [5]. This approach has shown promising, results with many studies demonstrating significant improvement in cognitive score when opting for HA‐WBRT instead of WBRT [10, 11, 12].

One notable disadvantage of this technique remains the peri‐hippocampal metastasis in the hippocampal avoidance area, which raises a risk–benefit dilemma when deciding on proper management of brain metastasis. We report a case of a patient who developed rapid intracranial progression with a single lesion in the brainstem after HA‐WBRT that was successfully salvaged with reirradiation.

## 2 Case

**FIGURE 1:**
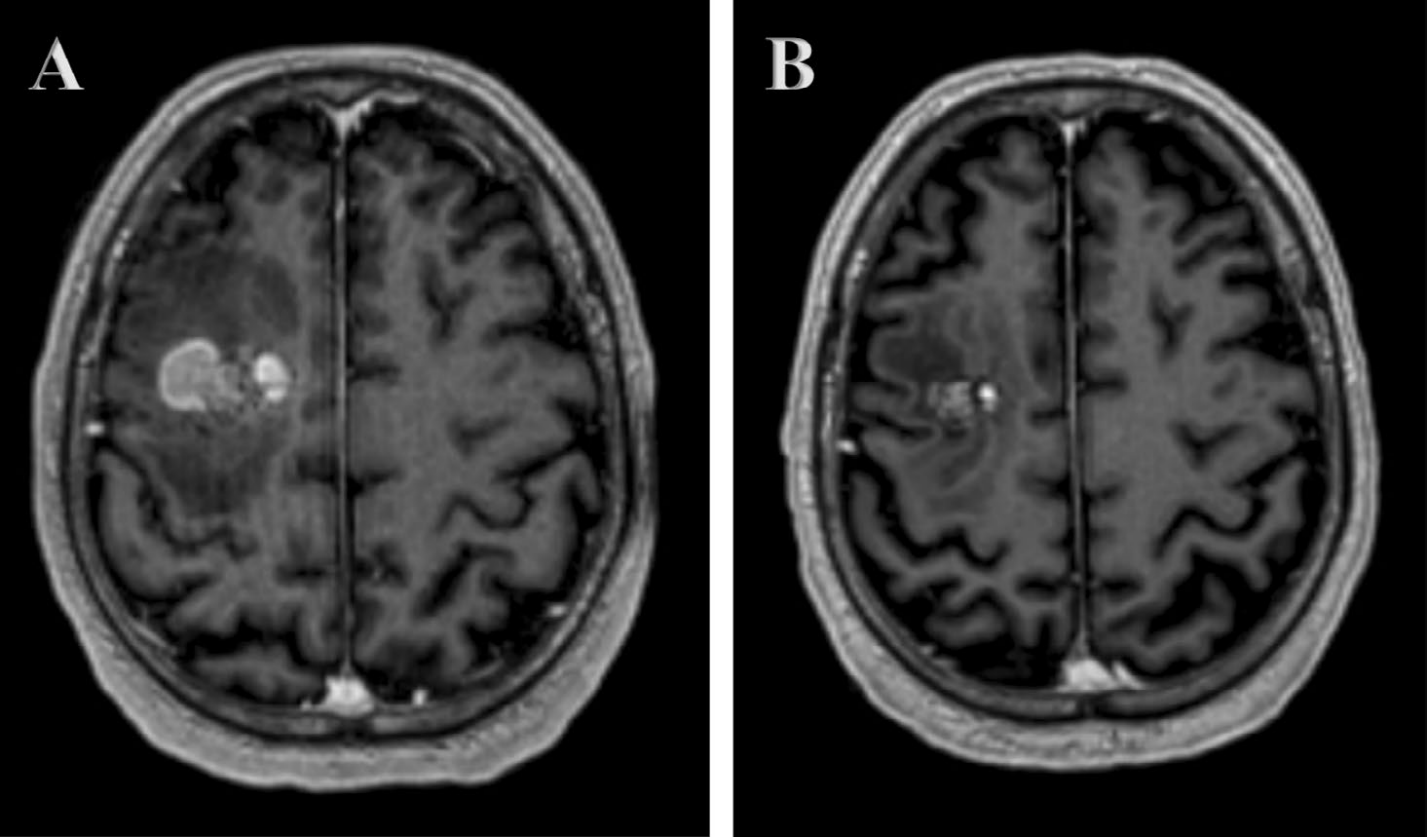
Axial T1‐weighted brain MRIs showing (A) supratentorial rim‐enhancing hemorrhagic lesion in the right middle frontal gyrus with marked surrounding vasogenic edema and (B) decrease in the size of the lesion with some increase in the surrounding vasogenic edema.

**FIGURE 2:**
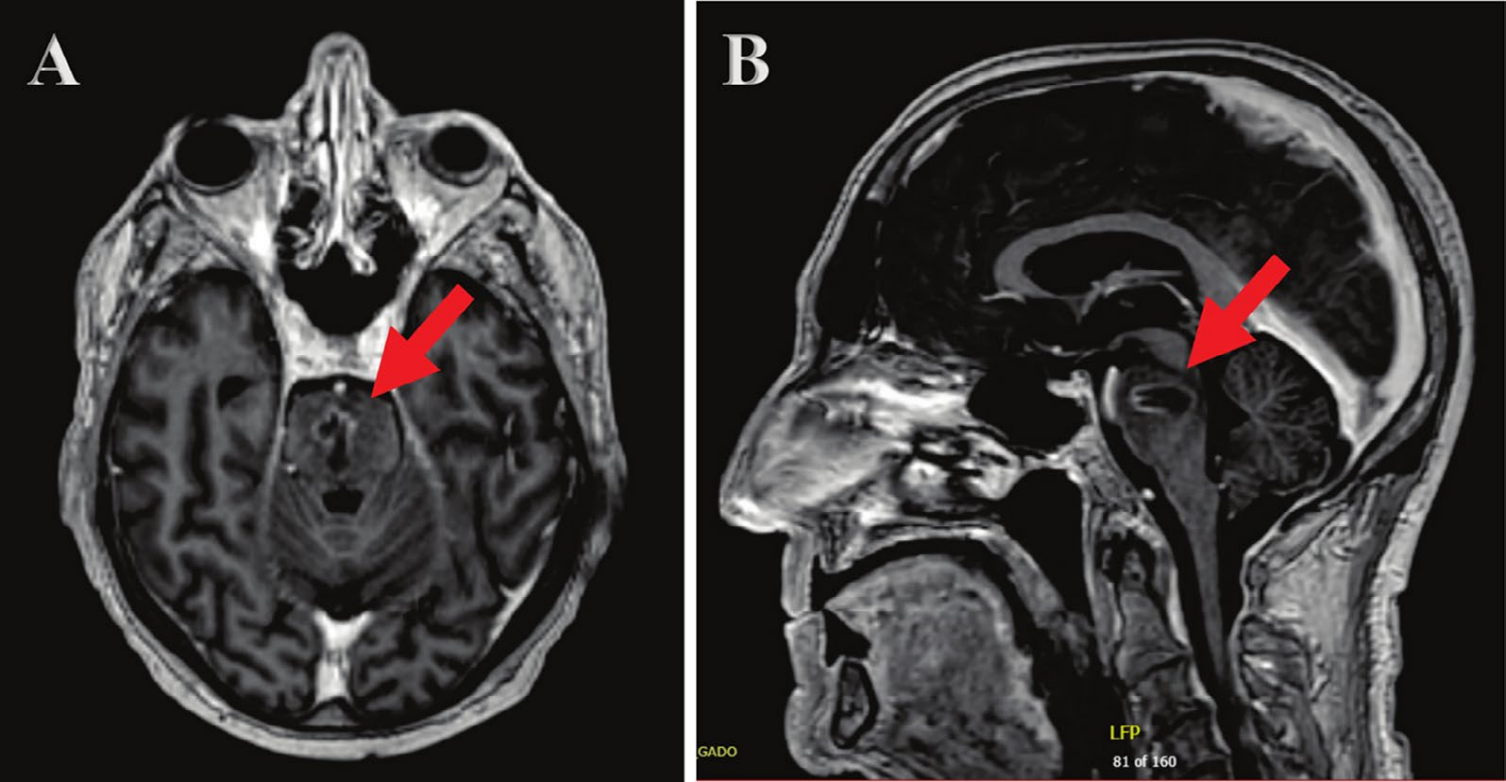
Axial (A) and sagittal (B) T1‐weighted brain MRIs showing a lesion containing hemosiderin with peripheral enhancement in the central aspect of the pons, associated with surrounding vasogenic edema.

A 73‐year‐old male presented in April 2019 to the Emergency Department in the American University of Beirut Medical Center (AUBMC) for a simple partial seizure, which subsided after starting antiepileptics and corticosteroids. Brain CT findings were highly suggestive of metastasis, which was confirmed by brain MRI showing nine supratentorial rim‐enhancing lesions, hemorrhagic in nature, the largest in the right frontal lobe with marked surrounding vasogenic edema. Additional imaging included a chest CT showing a right lower lobe spiculated mass with foci of enhancement consistent with a primary lung malignancy, and an abdomen/pelvis CT showing two lytic lesions in the left acetabulum and T9 thoracic vertebra, which were also consistent with metastasis. Core needle biopsy of the right lung mass proved the primary tumor was an invasive moderately differentiated lung adenocarcinoma with 10% programmed death‐ligand 1 (PD‐L1) positivity. Further genetic profiling revealed that the tumor was negative for EGFR and ALK mutations, but it did have an NTRK3 nonfusion gene mutation. After coordinating with the medical oncologists, the radiation oncology team decided on hippocampal avoidance whole brain radiotherapy (HA‐WBRT) as the best approach to treat the multiple brain lesions while also minimizing the neurocognitive sequelae. Radiotherapy was given in May 2019 as intensity‐modulated radiotherapy (IMRT) 30 Gy in 10 fractions along with memantine which was taken for 6 months. The treatment went smoothly with no adverse events reported by the patient; however, 2 months after finishing his radiotherapy sessions he presented again to our Emergency Department for a 2‐day history of progressive worsening headache associated with binocular horizontal diplopia and some gait imbalance. Brain MRI done showed significant improvement in baseline lesions (Figure 1), with interval decrease in the size of the heterogeneously enhancing hemorrhagic lesions in the right middle frontal gyrus measuring 1.5 × 1.2 cm, previously measuring 3.1 × 2 cm. In addition, there was an interval decrease in the size of the left cingulate gyrus lesion measuring 0.9 cm, previously 1.3 cm. Some decrease was noted in the size of the left parietal lesion with no significant change in the size of the punctate enhancing lesions in the right anterior frontal and left posterior temporal lobes. On the other hand, there was an interval appearance of a new lesion containing hemosiderin with peripheral enhancement in the central aspect of the pons measuring 2 × 1.1 cm, associated with surrounding vasogenic edema extending to the level of the cerebral peduncles, confirming a new metastatic lesion (Figure 2).

**FIGURE 3:**
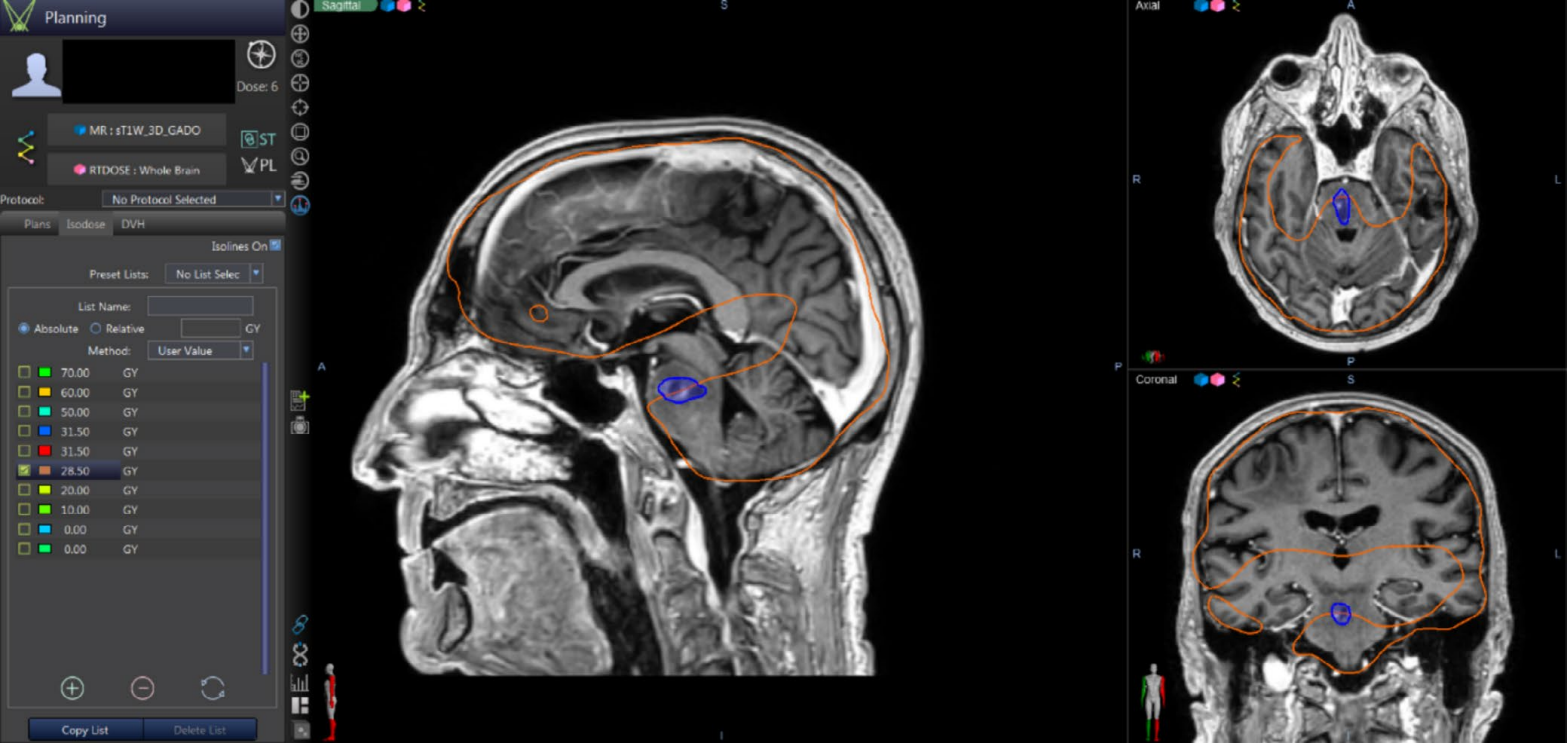
Fusion image of the intensity‐modulated radiotherapy (IMRT) plan of hippocampal avoidance whole brain radiotherapy (HA‐WBRT) showing 95% of the prescription dose (in orange) with the T1‐weighted brain MRI showing the new pontine lesion (in blue).

To better interpret this fast‐occurring metastasis, the HA‐WBRT plan received by the patient was fused with the brain MRI shown in Figure 2. The location of the new brainstem lesion was found to be in a partially cold area located around 19 mm away from the hippocampi (Figure 3), and receiving minimum and mean doses of 23.8 Gy and 27.84 Gy, respectively, which are both lower than the prescription dose of 30Gy. This could have contributed to the occurrence of the new lesion in this specific location in spite of the impressive response seen in all the other baseline metastatic lesions.

The patient was subsequently scheduled for stereotactic radiotherapy (SRT) of 21 Gy in 3 fractions to the new brainstem metastasis, to be taken with dexamethasone 8 mg tapered over 3 weeks. In addition, he received systemic therapy with Carboplatin, Pemetrexed, and Pembrolizumab as part of his initial treatment plan.

On December 2019, 4 months after finishing the SRT sessions, the patient returned to the hospital with a positional headache and a subjective feeling of falling to the right, associated with right facial weakness. Brain MRI followed by MR spectroscopy, diffusion, and perfusion showed evidence of radiation necrosis of the treated brainstem metastasis. This was well managed with Dexamethasone taken as 8 mg twice daily, tapered over 5 weeks, along with the addition of Bevacizumab.

He lasted 2 months before seeking our care again in March 2020 due to new onset of fever and seizures. Brain CT at that time ruled out any intracranial progression or new brain metastasis, so further workup included a lumbar puncture and an electroencephalogram (EEG) that confirmed the diagnosis of HHV‐6 encephalitis. He was treated with Gancyclovir for 21 days, in addition to antiepileptics, leading to good clinical improvement.

The patient's last follow‐up was on August 2020 when he presented to the ED for pneumonia and UTI, with Chest CT showing unchanged right lower lobe soft tissue mass and abdomen/pelvis CT showing unchanged multiple sclerotic and lytic metastatic osseous lesions. He was lost to follow‐up afterwards.

**FIGURE 4:**
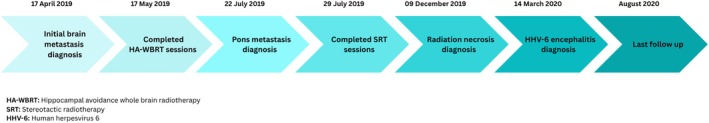
Timeline of events from initial diagnosis to last follow up.

Figure 4 summarizes the patient's flow of events from initial diagnosis to the last follow up.

## 3 Dosimetric Plan

We opted to compare the patient's old HA‐WBRT plan, which was designed using the Prowess software version 5.1 and delivered through IMRT, with a similar plan delivered through volumetric‐modulated arc therapy (VMAT), known for its improved dose conformity and treatment efficiency, to test its potential in resolving the drawback of brainstem undercoverage seen in this patient's fusion image (Figure 3).

**FIGURE 5:**
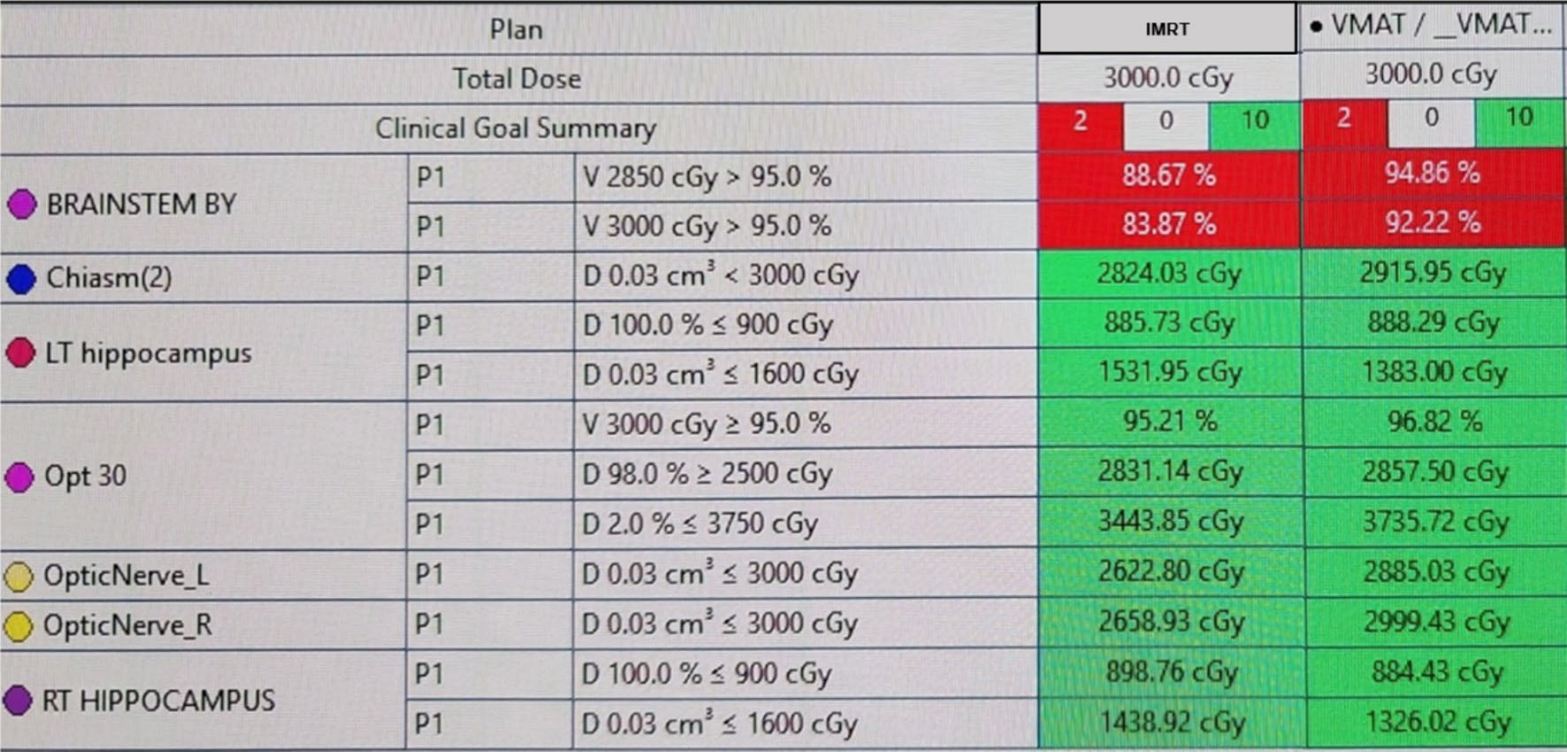
Clinical goal summary of the original IMRT plan vs. the new VMAT plan (in green: values meeting dose constraints, in red: values failing to meet dose constraints).

Both the original and new HA‐WBRT plans were developed in accordance with the RTOG 0933 protocol, with all relevant organs‐at‐risk (OAR) contoured, including the bilateral hippocampi, optic nerves, and optic chiasm, and a 5 mm isotropic margin applied around both hippocampi to define the hippocampal avoidance region (HAR) used to limit hippocampal dose exposure. Although the brainstem is not included as an OAR in the RTOG 0933 protocol, it was contoured in this study to evaluate the dose it received during treatment planning. Eclipse software version 18.0.1 was used to design, optimize, and evaluate all new radiation treatment plans in this dosimetric analysis. Figure 5 shows a dosimetric table comparing the clinical goals achieved in the IMRT and the VMAT plans.

Both IMRT and VMAT plans met all protocol‐specified dose constraints, with minor differences noted in OAR sparing. Unfortunately, neither plan achieved optimal brainstem coverage. In the IMRT and VMAT plans, 95% of the prescribed dose (28.5 Gy) reached 88.67% and 94.86% of the brainstem volume, respectively, indicating suboptimal coverage relative to standard therapeutic goals (≥ 95% of the PTV should receive ≥ 95% of prescribed dose). In addition, when evaluating the volume of brainstem receiving 100% of the prescribed dose (30 Gy), the IMRT plan covered 83.87% of the brainstem, while the VMAT plan covered 92.22%.

These findings suggested that while both plans are compliant with hippocampal avoidance protocol standards, additional optimization would be necessary to adequately achieve brainstem coverage.

**FIGURE 6:**
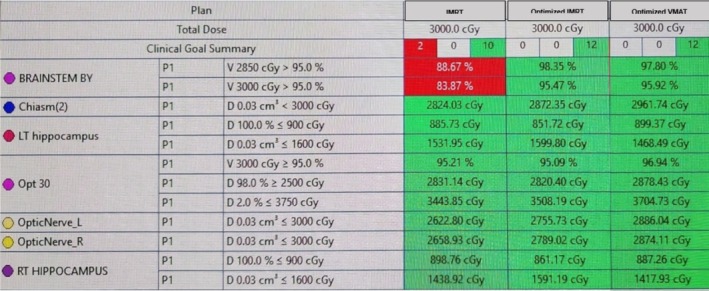
Clinical goal summary of the original IMRT plan vs. the new optimized IMRT and VMAT plans (in green: Values meeting dose constraints, in red: Values failing to meet dose constraints).

Subsequently, we designed modified HA‐WBRT plans to optimize dose coverage of the brainstem, such that 95% of the brainstem volume receives at least 95% of the prescribed dose. This was done by incorporating the brainstem into the optimizer as a planning objective, while still considering the hippocampal avoidance and OAR constraints. Two plans were created across the two treatment techniques: IMRT and VMAT. Figure 6 shows a dosimetric table comparing the clinical goals achieved in the original IMRT plan vs. the optimized IMRT and VMAT plans.

As illustrated in Figure 6, all three plans met the RTOG 0933 protocol dose constraints for the hippocampi and designated OARs. However, following brainstem dose optimization, 95% of the prescribed dose covered 98.35% of the brainstem in the IMRT plan and 97.80% in the VMAT plan, indicating numerically adequate coverage in both. Additionally, the full prescription dose (30 Gy) covered 95.47% of the brainstem in the optimized IMRT plan and 95.92% in the optimized VMAT plan.

**FIGURE 7:**
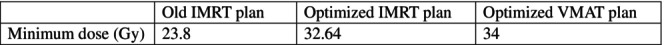
Minimum dose reaching the new pontine lesion in the 3 treatment plans: Old IMRT, optimized IMRT, and optimized VMAT.

Furthermore, we compared the minimum dose reaching the new pontine lesion across the 3 treatment plans and noticed a significant numerical increase in the optimized plans when compared to the original IMRT plan (Figure 7).

This dosimetric analysis supports the feasibility of achieving acceptable brainstem coverage in HA‐WBRT plans through brainstem optimization, without compromising OAR constraints.

## 4 Discussion

WBRT was considered the mainstay of treatment of brain metastasis for several decades. Nowadays, it is replaced, whenever indicated, by novel modalities that have less neurocognitive effects and better QOL [12, 13]. As an attempt to decrease the toxicity of WBRT, many protocols were designed, including lowering the WBRT doses or adding neuroprotective agents [14]. The majority of those trials failed. One of the few exceptions is the use of Memantine that achieved an improvement in terms of neurocognitive function when added to WBRT [15]. Other trials deferred the use of WBRT in favor of stereotactic radiosurgery, which demonstrated comparable survival outcomes with a better neurocognitive profile [16, 17, 18], making SRS the current standard of care in patients with limited intracranial disease, newly defined as having up to 10 brain lesions with total cumulative volume not exceeding 15 mL [19].

Another protocol that showed impressive results in neurocognitive preservation was the hippocampal avoidance whole brain radiotherapy (HA‐WBRT). HA‐WBRT gained popularity around 2010 after the phase II RTOG‐0933 trial demonstrated that a reduced radiation dose to the hippocampal neural stem‐cell compartment was associated with a significantly smaller decline in recall compared with a historical control. This reduction in hippocampal irradiation was evaluated in terms of cognitive preservation, using the Hopkins Verbal Learning Test–Revised Delayed Recall (HVLT‐R DR), which compared memory performance between HA‐WBRT and WBRT patients. Results of the HA‐WBRT arm showed a mean relative decline of 7% from baseline to 4 months, which was significantly lower than the historical control of a 30% decline. QOL results were also improved when compared to WBRT alone [10].

This outcome was further validated by the phase III NRG‐CC001 trial which compared the effect of WBRT with memantine, with or without hippocampal avoidance. Results showed that despite no significant difference in survival outcomes between the 2 arms, the risk of cognitive failure and patient‐reported neurologic symptoms was significantly lower in the hippocampal avoidance arm [20, 21]. These results gave rise to a new standard treatment for patients with multiple brain metastases not amenable to SRS. HA‐WBRT plus Memantine is now recommended for patients with good performance status, who have no tumor in or within 5 mm of the hippocampus and have at least 4 months of expected survival [5].

The hypothesis behind this anatomical avoidance technique arose in the light of clinical and preclinical evidence suggesting the presence of neural stem cells in the hippocampus, specifically the dentate gyrus, responsible for neurocognitive characteristics. These cells were proven to be sensitive to cranial irradiation that increases their rate of apoptosis while also decreasing their neurogenesis [22, 23, 24, 25, 26, 27].

The main concern regarding this technique is the probability of developing new brain metastases near the hippocampus, especially in the hippocampal avoidance (HA) region, which constitutes the hippocampus plus a 5 mm margin. Possible occult metastatic lesions in this region that receive a nontherapeutic dose of radiation make it a sensitive area for future recurrence or new metastasis, commonly referred to as peri‐hippocampal failure [28].

One recent systematic review which evaluated this risk of relapse was Leskinen et al. who included 11 related studies, all of which consistently showed low peri‐hippocampal failure rates in HA‐WBRT patients, with a combined rate of 4% as per a random effects model, in addition to impressive neurocognitive testing results 3–24 months post‐treatment. It also included 5 studies reporting no significant difference in relapse rates when compared to conventional WBRT [29]. Similarly, Wiegreffe et al. included 40 records and 5374 patients in their systematic review, which also concluded that HA‐WBRT trials had similar peri‐hippocampal failure rates to the conventional WBRT trials, with an overall rate of 4.5% after hippocampal avoidance prophylactic cranial irradiation (HA‐PCI) and HA‐WBRT, most of which were deemed salvageable with radiosurgery [30].

Our patient was treated with HA‐WBRT for 9 brain metastases originating from a primary NSCLC, which resulted in a good response in the treated lesions, as seen on a follow‐up MRI done 2 months post‐radiotherapy. However, the same MRI showed a new solitary lesion in the upper aspect of the pons, which is located in close proximity to both hippocampi. While peri‐hippocampal failure is a recognized risk post HA‐WBRT, the occurrence of this relapse raises several questions concerning its cause, since no data have been documented yet demonstrating such a rapid relapse in a single area of the brain that was previously uninvolved.

Several studies reported isolated cases of peri‐hippocampal failure, most of which occurred between 4 and 22 months post HA‐WBRT [28, 31, 32, 33]. To our knowledge, the earliest recurrence reported so far was that of a 60‐year‐old patient with stage N3 small cell lung cancer SCLC who developed intracranial failure 3 months after HA‐PCI [34].

According to the literature, the standard incidence of brain metastasis within the hippocampal avoidance region ranges between 3.3% and 12.2%, while not much data is present regarding the areas further away from the hippocampus, which meet the brainstem [30, 35, 36, 37, 38]. One study reported a 2.6% risk of brainstem metastasis when reviewing images of 100 patients with known brain lesions [35], while another study including 632 patients described an incidence of 8.4% in the area 20 mm away from the hippocampus [36], which is close to the estimated distance between the hippocampus and the new lesion (19 mm). This rate of peri‐hippocampal metastasis can be further increased by many risk factors.

One possible factor is the primary tumor's aggressiveness or predisposition to peri‐hippocampal metastasis. NSCLC tumors metastasizing to the brain are very familiar in the literature. One retrospective study, which reviewed the records of 335 NSCLC patients, reported a peri‐hippocampal metastasis incidence of 9%; however, univariate analyses did not show any significant association between the latter and tumor type [39]. On the other hand, a multi‐variable analysis performed by Ahn et al. revealed a significant association between extracranial metastasis and peri‐hippocampal metastasis when reviewing 123 NSCLC patients records [40], which may explain our patient's rapid relapse, considering he had several bone metastasis foci upon initial diagnosis.

Another factor to account for when interpreting peri‐hippocampal metastasis should be the primary tumor's molecular markers. EGFR and ALK rearrangements are known risk factors of brain metastasis in NSCLC [41, 42, 43] ever not much literature is present regarding their role in hippocampal and peri‐hippocampal metastasis. Ahn et al. did not find a significant association between EGFR, ALK, and ROS1 rearrangements and the incidence of peri‐hippocampal metastasis; however, these results were limited by the scarcity of subjects [40], necessitating further research on this matter.

Several other factors have been proven to play a role in increasing the risk of peri‐hippocampal involvement. Gondi et al. retrospectively analyzed several variables in patients with ≤ 10 brain metastases, and upon binary logistic regression showed that only the aggregate volume of intracranial metastases was a statistically significant predictor of metastasis in the HA region, further demonstrating that volumes of 1, 10, and 35 cm^3^ had an estimated incidence of peri‐hippocampal disease of 6.4%, 7.5%, and 11.8%, respectively [37]. In our case, the estimated aggregate volume of intracranial metastases extrapolated from the radiology report of the initial brain MRI was around 9.1 cm^3^, placing our patient at an approximately 7.5% risk of involvement in the HA area.

A similar retrospective study applied multivariate logistic regression using additional risk factors and compared the risk of metastasis within several hippocampal margins, including a 20 mm margin, demonstrating that the number and volume of brain metastases were both significantly correlated with the latter [44]. Patients with 4 or more brain lesions had 5 times the risk of peri‐hippocampal disease within the 20 mm radius, which poses a higher threat than what was previously reported by Han et al., who estimated a 3.84 times higher risk of brain metastasis in this region when harboring ≥ 6 brain lesions [45]. This study also had a different take on the cutoff for the total volume of metastases, as a volume above 4457mm^3^ had a 4‐fold increase in the risk of metastasis 20 mm within the hippocampus. Both these risk factors place our patient in a high‐risk group for peri‐hippocampal failure.

As for causes that are not tumor‐related, underdosing of the upper brainstem seems to be a common and inherent occurrence in HA‐WBRT plans. In a dosimetric study done by Zhang et al. on 20 patients receiving 30 Gy/10 HA‐WBRT that compared helical tomotherapy (HT) with volumetric modulated radiation therapy (VMAT), the average minimum dose received by the brainstem was 15.79 ± 5.86 Gy and 17.33 ± 3.58 Gy, respectively [46], which are both considerably lower than the RTOG 0933 dose constraint of PTV D_98%_ ≥ 25 Gy. Similarly, several studies reported underdosing of the peri‐hippocampal area when adopting IMRT planning as well [34, 47].

Upon reviewing our case's old radiotherapy plan, it was confirmed that the location of the pontine metastasis was underdosed (Figure 3), which showed a minimum dose of 23.8 Gy.

Although IMRT has long been proven to provide highly conformal dose distribution, it has shown some limitations in terms of hippocampal sparing and meeting the RTOG 0933 constraints, owing to its beam angle limitations and lower flexibility when it comes to controlling dose falloff around the hippocampus [34, 47, 48]. VMAT and helical tomotherapy (HT) modalities offer rotational intensity modulation which has gained them superiority in the HA‐WBRT technique, resulting in better dose uniformity and hippocampal sparing when compared to IMRT [48, 49]. VMAT treatment planning specifically has been known to spare the hippocampus while providing both excellent target coverage and fast delivery, with some studies showing slight preference for non‐coplanar VMAT plans [50, 51, 52, 53].

Based on these findings, we compared our patient's original HA‐WBRT IMRT plan to a VMAT‐based one to assess whether the latter could improve brainstem coverage while maintaining compliance with the RTOG 0933 protocol. Although both plans met all protocol dose constraints, neither achieved optimal brainstem coverage, with < 95% of the brainstem receiving 95% of the prescribed dose. To address this, new HA‐WBRT plans were designed using IMRT and VMAT to include brainstem coverage as an optimization goal. The optimized IMRT and VMAT plans successfully improved brainstem coverage to 98.35% and 97.80%, respectively, with increased volumes receiving the full prescription dose as well. Both optimized plans continued to meet RTOG 0933 constraints as well.

## 5 Conclusion

Our results demonstrate that targeted brainstem optimization can substantially improve brainstem dose coverage in HA‐WBRT plans without violating critical OAR constraints. Given the clinical significance of brainstem metastases and their potential to cause severe neurological symptoms that impact patients' quality of life, future prospective studies are warranted to validate these results in a larger patient population and to assess their clinical relevance.

## Author Contributions

Conceptualization: Zeinab Dandash, Bassem Youssef, Lara Hilal, and Toni Tannoury. Writing – original draft: Zeinab Dandash, Bassem Youssef, Lara Hilal, and Ali Al‐Zein. Writing – review and editing: Zeinab Dandash, Bassem Youssef, Lara Hilal, Ali Al‐Zein, and Arafat Tfaily. Supervision: Bassem Youssef and Lara Hilal. All authors had full access to the data, contributed to the study, approved the final version for publication, and took responsibility for its accuracy and integrity.

## Ethics Statement

Ethical approval for this case report was waived in accordance with the Human Research Protection Program (HRPP) at the American University of Beirut (AUB), which does not require IRB approval for single‐patient case reports that do not involve identifiable data or experimental interventions.

## Consent

We had previously obtained the patient's written consent for the publication of any potentially identifiable images or data included in this article, and there is no personal information regarding the patient in this case report.

## Conflicts of Interest

The authors declare no conflicts of interest.

## Data Availability

The data that support the findings of this study are available on request from the corresponding author. The data are not publicly available due to privacy or ethical restrictions.
